# Evaluating the Effectiveness of the Third Molar Maturity Index (I_3m_) and Mandibular Condyle Cortication for Determining the Legal Age (18) of Latvian Individuals

**DOI:** 10.3390/diagnostics15040475

**Published:** 2025-02-16

**Authors:** Zanda Bokvalde, Liene Zamure-Damberga, Maksims Zolovs, Laura Neimane

**Affiliations:** 1 Department of Conservative Dentistry, Institute of Stomatology, Riga Stradins University, LV-1007 Riga, Latvia; 2 Department of Conservative Dentistry and Oral Health, Riga Stradins University, LV-1007 Riga, Latvia; 3 Statistics Unit, Riga Stradins University, LV-1007 Riga, Latvia; 4 Institute of Life Sciences and Technology, Daugavpils University, LV-5401 Daugavpils, Latvia; 5 Department of Diagnostic Radiology, Institute of Stomatology, Riga Stradins University, LV-1007 Riga, Latvia

**Keywords:** dental age estimation, dental panoramic radiograph, forensic odontology, mandibular condylar growth, third molar maturity index (I_3m_)

## Abstract

**Background**: It is important to accurately determine the legal age at which a person is considered and treated as an adult; in many countries, it is 18. With the increasing migration flow to European countries, accurate age estimation methods must be implemented. In this study, the third molar maturity (I_3m_) index and mandible condyle cortication stage were tested to distinguish adult from non-adult Latvian individuals using dental panoramic radiographs. **Methods**: This study included 716 selected dental panoramic radiographs of patients between the ages of 14 and 22. The lower-left third molar apical root parts were analyzed, and the I_3m_ index was calculated. In addition, the condyle cortication stage was evaluated. **Results**: All logistic regression models achieved statistically significant results (*p* < 0.001). The accuracy was high for all groups (males: 0.90, females: 0.87, both genders: 0.89), and the sensitivity was lower than the specificity, particularly for females (sensitivity: 0.55). The I_3m_ index appears to be a strong predictor across all models, while the mandibular condyle cortication stage plays a more nuanced role, depending on sex and the stage of condyle maturation. A higher I_3m_ index value (greater than the cut-off of 0.095 in males) indicates a higher likelihood of being classified as a non-adult male for this model; however, in females, a cut-off point higher than 0.150 indicated a higher likelihood of being classified as a non-adult female. The new proposed cut-off values need to be tested on a new sample. **Conclusions**: The I_3m_ index is a reliable age estimation tool, and a modified cut-off value could be determined for each gender in Latvian individuals. The condyle cortication stage is a weak tool for chronological age estimation in dental panoramic radiographs.

## 1. Introduction

Age estimation is performed for various reasons. For cadavers, age estimation is performed during criminal cases as well as for victims of mass disasters, fires, crashes or homicides. Age determination on living persons may be performed in criminal proceedings cases or in the case of undocumented immigration, when a birth certificate is not present, or when the responsible authorities have a reason to believe that documents are falsified [1,2]. It is also important to accurately determine the legal age at which a person is considered and treated as an adult; in many countries, it is 18.

According to the Study Group on Forensic Age Diagnostics of the German Association of Forensic Medicine (AGFAD), the age estimation examination consists of dental and physical analyses, a dental panoramic radiograph, and an X-ray of the left hand or an additional examination of the clavicles if the skeletal development of the hand is complete [2]. Therefore, radiology plays a significant role in age estimation, in which radiographs of teeth and bones are analyzed. Tooth development is considered a steady process controlled more by genes, but it is little affected by nutritional, hormonal, or pathological changes; therefore, it can play an important role in age estimation [1]. Teeth are the strongest tissues of the human body and can withstand serious external factors. Of particular importance for age estimation is the eruption and mineralization of the third molar. For evaluation of the third molar, the previously mentioned dental panoramic radiograph is used, which gives a greater insight into the state of the dentition and surrounding hard tissues [2,3].

In European countries, especially in western Europe, migration flow has increased remarkably in recent decades, and migration has peaked in the last decade. In 2022, EU countries received 873,700 requests for asylum. In 2023, there was an increase in incongruous border crossing. In 2023, there were 1,048,900 asylum seeker applications, and around 24% of these requests were from individuals under the age of 18. The asylum seekers were mainly Syrian, Turkish, Afghan, Venezuelan, and Columbian, and there were more male applicants than female [4]. However, in Latvia, increased migration flow has only been observed in the past few years. Increased numbers of asylum seekers from third world countries have been trying to cross the eastern border of Latvia, which is also the eastern border of European Union, illegally for the past years, creating chaos in the structure of interior affairs [5]. Some of these foreigners cannot or choose not to present any documents confirming their date of birth. For this reason, alternative and reliable methods are needed to confirm whether the person is under 18. To perform chosen age estimation methods accurately, forensic odontology specialists must be accessible, which is not achievable in many countries due to a lack of forensic odontology training for dentists [6]. A study from Wadhwan et al. (2014) about forensic odontology education mentions the incompetence of general dentists when it comes to age estimation [7]. Accordingly, general dentists need to be educated about age estimation methods as there seems to be a growing necessity.

Around the age of 18, most teeth have erupted and matured except for the third molars. Third molar development takes place from 16 to 23 years of age [8,9]. There are various methods described in the literature that involve analyzing tooth mineralization stages or tooth eruption/root formation [1]. A systematic review and meta-analysis performed by Marconi et al. (2022) investigated different age estimation methods [10]. In their review, they concluded that Demirjian’s method tended to overestimate chronological age for both genders, but Nollas’ method and Cameriere’s methods might be recommended as more preferred approaches for age estimation, although both methods need further research [10]. In 2008, Cameriere et al. introduced a method for dental age estimation, also referred to as the third molar maturity index (I_3m_), where measurements of the width of the lower left third molar apices and the height of the tooth are made. The I_3m_ index uses a specific cut-off value of 0.08 and shows favorable results in discriminating adults from non-adults [11].

Mandible condyle development is associated with age. The formation of the condyle starts during the ninth intra-uterine week and continues until age 22. A homogenous bony layer on the mandibular condyle periphery starts to form around age 12–14. Until age 22, bone formation can be seen in different cortication stages [12,13]. The mandibular condyle cortication stage increases with age, and a few studies have examined the possible relationship between age estimation and condyle cortication stage, suggesting the potential use of this method to estimate age [12,14,15].

The aim of this study was to test the I_3m_ index and mandible condyle cortication stage for their ability to distinguish adult from non-adult Latvian individuals using dental panoramic radiographs. The null hypothesis stated that the I_3m_ index and condyle cortication stage has no validity in detecting the legal age of 18 years in Latvian individuals on dental panoramic radiographs.

## 2. Materials and Methods

### 2.1. Case Selection

This retrospective study was conducted in accordance with the Declaration of Helsinki and received approval from the Ethics Committee of Riga Stradins University (2-PĒK-4/535/2022). The study consisted of 716 selected dental panoramic radiographs of individuals (474 females and 242 males) between the ages of 14 and 22. Table 1 shows age and gender distribution. Although ethnicity was not documented in this study, the sample mainly consisted of individuals of Latvian origin. The radiographs were taken in the Riga Stradins University Institute of Stomatology (RSU SI) clinic between January 2018 and July 2018. Selected radiographs were taken for various different clinical purposes, and none were prescribed for the study. Data from the patient’s charts about their health status were not recorded, as this was not the main purpose of this study.

The radiographs were obtained with a Veraview 800X scanner, Morita, Japan, and acquired from the database of RSU SI. The acquired radiographs were analyzed using i-Dixel imaging software, Version 2.15.0 (Morita, Osaka, Japan), on a medical monitor (CR240G, Jusha, Nanjing, China). In order to perform the I_3m_ index measurements and to evaluate the mandibular condyle, both the brightness and contrast were adjusted on the radiographs; additionally, the zoom-in function was used to examine the area of interest.

The exclusion criteria are listed in Table 2 and were as follows: low image quality or an incomplete radiograph, a missing or extracted lower-left molar or an abnormal root morphology (only third molars with 2 roots were included), signs or the presence of TMJ disorders (such as arthritic changes, trauma), signs or suspicion of genetic disorders, pathologic situations that affected the area of interest, such as a dentigerous cyst or periapical inflammation of the third molar, and anatomical structures that overlapped the area of interest.

Due to different exclusion criteria, some of the dental panoramic radiographs were evaluated only for the I_3m_ index but not condyle maturation, and vice versa. For the I_3m_ index, 539 dental radiographs were included, and for the mandible condyle maturation stage, 665 dental radiographs were included.

### 2.2. Measurements and Data Analysis

**Figure 1:**
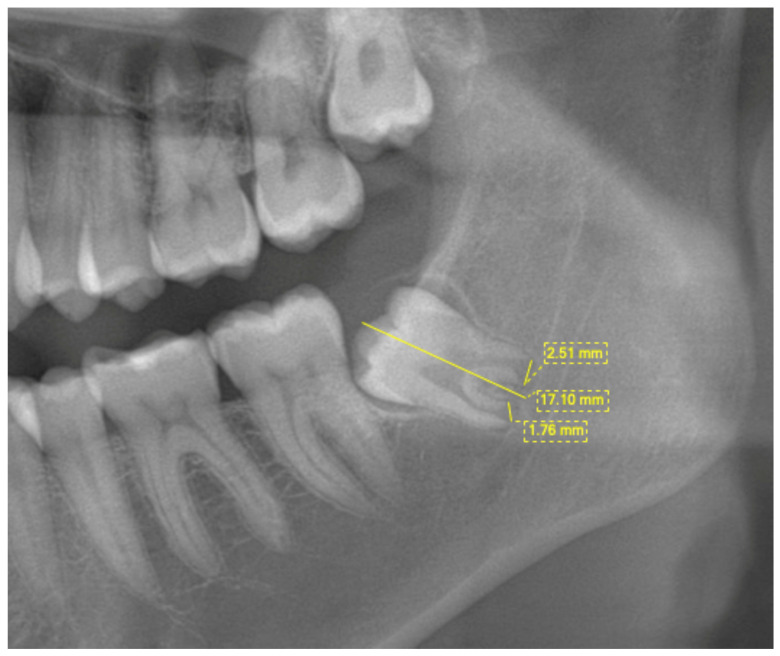
I_3m_ measurements of the lower-left third molar.

The I_3m_ index was calculated according to Cameriere et al. [11]. The lower-left third molar apical root parts were analyzed, and the I_3m_ index was calculated. The index is evaluated as the sum of two measurements of the inner sides of the apical root ends, which are then divided by the tooth length, as shown in Figure 1. The obtained ratio then allows for the use of the I_3m_ index. When the I_3m_ index was 0.08 or more, the individual was considered to be below the age of 18 years. On the other hand, if the I_3m_ index was less than 0.08, then the individual was considered to be at least 18 years old. If root development was complete, then I_3m_ = 0 and the individual was considered older than 18.

**Figure 2:**
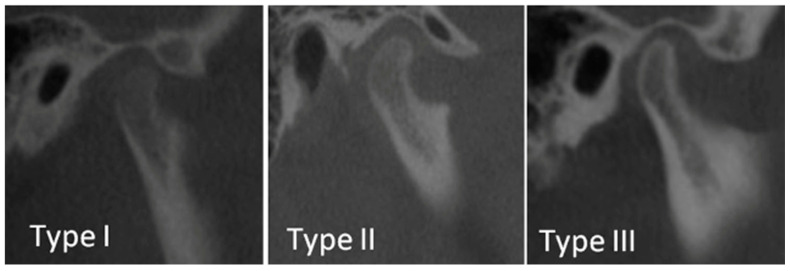
Type 1, 2, and 3 cortication stages of mandibular condyle in CBCT images [14].

The evaluation of condyle maturation was carried out according to a study by Bayrak et al. [14], in which this method was applied to cone beam computed images (CBCT). The classification suggests 3 types of condyle maturation, as shown in Figure 2:

Type 1: No cortication observed on the condylar surface.

Type 2: Slight condylar surface cortication observed, but at a lower density than the surrounding bone structures.

Type 3: The condylar surface appears high-density and is easily visualized compared to the surrounding bony structures.

In this study, the method used for CBCT images was adopted and applied for panoramic images, understanding that the informativeness of radiological methods differ.

Radiographs where both condyles were well visualized were included in this study.

The first observer, a dentist with 8 years of experience (Z.B.), completed all evaluations of the radiographs blinded to the patient’s age and gender. After 2 weeks, every 10th radiograph was re-evaluated by the first observer, and the same selected radiographs were evaluated by a second observer, a dentist with 11 years of experience (L.Z.D.), to test the intra-rater and inter-rater reliability.

### 2.3. Statistical Data Analysis

A binomial logistic regression analysis was conducted to investigate the effectiveness of the I_3m_ index and mandibular condyle cortication stage for differentiating between adult and non-adult individuals. To build the regression model, a stepwise forward method was used. All possible models were calculated. To select the best model, the Akaike Information Criterion (AIC) was used. Additionally, a receiver operating characteristic curve (ROC) analysis was performed with the area under the curve analysis (AUC value) to evaluate the performance of the regression model as a binary classifier. AUC values greater than 0.7 are considered good in terms of distinguishing between the two classes: adult and non-adult individuals. Youden’s index was used to identify the optimal cut-off point. The reliability of the measurements was assessed using intraclass correlation coefficients (ICCs) for both intra-rater and inter-rater reliability. Statistical data analysis was performed with Jamovi (v.2.3). The results were considered statistically significant when the *p* value < 0.05.

## 3. Results

Table 3 summarizes the fit statistics for the logistic regression models developed for males, females, and both genders combined. All models achieved statistically significant results (*p* < 0.001). The AUC values were the highest for males (0.95), followed by the combined model (0.92), and then females (0.89). These values suggest good performance for all the models, with the model for males demonstrating the best discrimination ability. The AIC values were the lowest for males (87.5), indicating a better relative fit compared to the other models.

**Figure 3:**
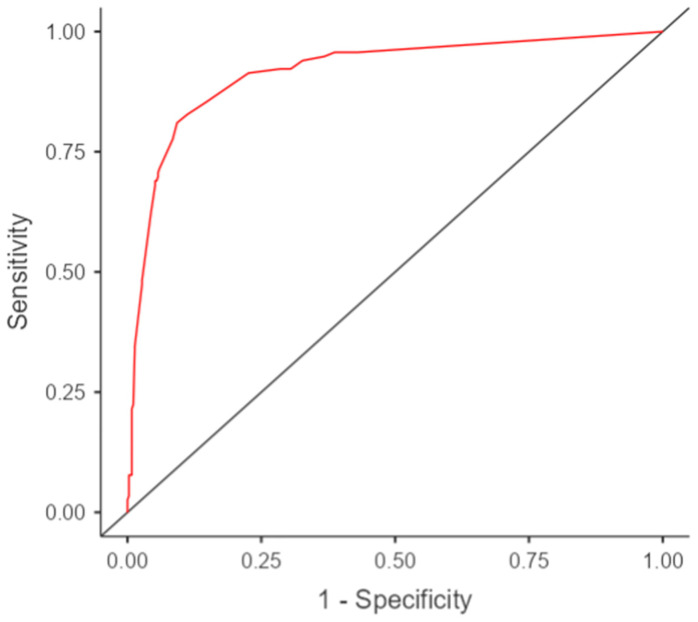
ROC curve indicating adult age by I_3m_ index in the sample for both genders.

The ROC curve (AUC) depicts the model’s ability to distinguish between adults and non-adults (Figure 3).

Table 4 presents additional metrics evaluating the models’ performance as binary classifiers. Accuracy reflects the overall proportion of correctly classified individuals (adult vs. non-adult). Specificity refers to the proportion of correctly classified adults, and sensitivity indicates the proportion of correctly classified non-adults. The models achieved high accuracy for all groups (males: 0.90; females: 0.87; both genders: 0.89), with slightly lower sensitivity compared to specificity, particularly for females (sensitivity: 0.55).

These results suggest that the logistic regression models effectively differentiate between adult and non-adult individuals. The I_3m_ index appears to be a strong predictor across all models, while the mandibular condyle cortication stage plays a more nuanced role depending on the sex and stage of the condyle maturation.

Table 5 details the estimated coefficients for each predictor variable within the logistic regression models. For all the models, the I_3m_ index had a positive and statistically significant coefficient (*p* < 0.001). This indicates that a higher I_3m_ index value is associated with an increased likelihood of being classified as a non-adult. In the male model, a 0.01 increase in the I_3m_ index value is associated with an 11% greater likelihood (95% CI: 6–17%) of being classified as a non-adult.

The condyle cortication coefficients varied depending on the model and condyle cortication type. The tables also report the odds ratio (OR) and 95% confidence interval (CI) for each predictor. These values can be used to quantify the effect size of the predictors on the model’s outcome. Finally, the cut-off point is presented, which represents the threshold value on the I_3m_ index used to classify individuals as adults or non-adults. A higher I_3m_ index value (greater than the cut-off of 0.095 in the male model) indicates a higher likelihood of being classified as a non-adult male for this model; however, in the female model, a cut-off point higher than 0.150 indicated a higher likelihood of being classified as a non-adult female.

**Figure 4:**
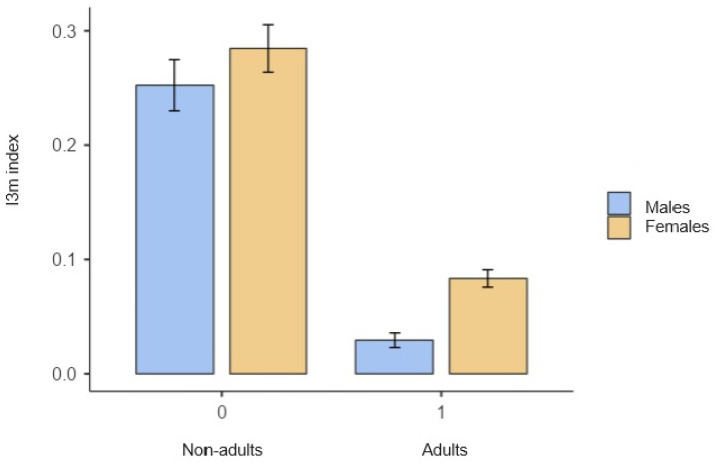
Relationship between the average I_3m_ index for each gender and age group.

Figure 4 shows the average I_3m_ index value for the non-adult and adult groups depending on gender. It shows how the values for females are higher, in general, than for men in both age groups.

The analysis of intra-rater reliability showed an intraclass correlation coefficient (ICC) of 0.97 (95% CI: 0.94–0.98), and the inter-rater reliability was ICC = 0.95 (95% CI: 0.91–0.97). This indicates a high level of reliability.

## 4. Discussion

Dental age estimation has not been widely studied in Latvia, and this is the first study of dental age estimation in Latvian individuals. According to the available literature, it is also the first study to test both the I_3m_ index and condyle maturation on the same radiographs in relation to chronological age. Our null hypothesis in this study for the I_3m_ index was rejected, but for the condyle cortication stage was approved, that is, the condyle cortication stage has limited use for detecting age on dental panoramic radiographs. The studies conducted so far show that the I_3m_ index has the potential to determine whether a person is an adult or a minor [11]. A meta-analysis from De Micco et al. (2021) [16] concluded that the I_3m_ index provides good results and has a slight risk of estimating age incorrectly. They mention that the index shows good results even in isolated populations, which are known for their genetic drift, with genetic modifications that are otherwise very rare in other populations [16,17]. In addition to using the I_3m_ index, it is possible to visualize the temporomandibular joint (TMJ) and evaluate the condyle maturation stage on the same dental panoramic radiograph to achieve greater insight into an individual’s growth [14].

The results confirmed the practicality of the I_3m_ index with a cut-off value of I_3m_ < 0.08. Slightly better accuracy was seen in males (0.90) than in females (0.87). Good results were achieved for specificity, which was almost equal between genders; however, the sensitivity was better in males (0.82) than in females (0.55). Overall, compared to other studies [11,18,19,20,21,22], the proportions of the results are similar except for sensitivity in females, which was much lower than in previously published studies on the I_3m_ index. The reasons for this difference in results could be similar to those in other studies: slower and later maturation in females [9,23,24,25,26]. In their study, AlQahtani et al. (2010) mention that females preceded males in tooth maturation until the age of 14, but after that age, males showed more progressive third molar maturation [8]. A recent study from Švabova et al. (2024) concluded that third molar maturation tends to complete earlier in males than in females, without mentioning the possible mechanisms [24]. A Lithuanian study from Trakiniene et al. (2019) reviewed the possible relationship between sex and third molar maturation and noted that answers tend to vary; some researchers deny any differences in tooth maturation between genders, while others confirm differences but cannot define plausible reasons [27]. In the same study, Trakiniene et al. (2019) conclude from their results that gender plays a significant role; for females, third molar maturation occurs much earlier (1.9 years in average) than in males [27]. The results of Balla et al. (2022) indicate that the underdevelopment of impacted third molars and a cut-off value of I_3m_ < 0.08 could lead to age misclassifications, but they did not encounter any major differences between genders [28]. Their findings suggest that impaction of the lower third molar could slow mineralization and therefore lead to an incorrect age estimation. The results from Balla et al. (2022) [28] could explain the low sensitivity in our female group, because females tend to have smaller jaws than males, leading to more frequent third molar impaction. There seems to be uncertainty and considerable disagreement about the potential reasons for the discrepancy between genders. Other published articles suggest that population-specific methods could be required for age estimation. Therefore, specific cut-off values could be used depending on the individual’s nationality and possibly gender [22,29,30,31].

In this study, the results of logistic regression models demonstrated adequate differentiation between adult and non-adult individuals. When the results were compared to other European studies, which tested different cut-off values, the majority stated that I_3m_ < 0.08 provided the best results for discriminating adults from non-adults [18,19,32,33,34,35]. Proposed alternative I_3m_ cut-off values, by other authors, are listed in Table 6. Różyło-Kalinowska et al. (2018) is the only European study to suggest using a different cut-off value of I_3m_ < 0.07. The study indicates that both cut-off values can be used. I_3m_ < 0.07 yields better results for specificity, but I_3m_ < 0.08 is better for classification [21]. A study on an Albanian sample [22] suggested that more research is needed for population-specific methods or cut-off values. Further, when the results are compared to studies conducted in other continents and studies that analyzed the cut-off value, the majority declared that I_3m_ < 0.08 provides the best results for discriminating between adults and non-adults [36,37,38,39,40,41,42]. Differences between ethnicities have been studied before [9,43] with divergent results, suggesting that ethnicity has a clinically negligible role in estimating age. A recent study from Balla et al. (2022) proposed a cut-off value of I_3m_ < 0.17, and they conclude that it showed improved accuracy and sensitivity compared to a standard cut-off value of I_3m_ < 0.08; however, the specificity decreased when the cut-off value was changed. They concluded that an improved cut-off value allows for fewer false negatives—individuals who are 18 or older, but are classified as minors. [44]. Results similar to ours were observed in Goetten et al. (2022), suggesting a different cut-off value for females [23]. They suggest that changing the cut-off value for females to I_3m_ < 0.12 improves accuracy, while for males, I_3m_ < 0.08 gives satisfactory results [23]. A study from Dardouri et al. (2016) mentioned using a cut-off value of I_3m_ < 0.09 to improve sensitivity for both genders [45]. A future study should be conducted in which the new suggested cut-off values of I_3m_ < 0.095 (males) and I_3m_ < 0.015 (females), obtained from our results, are applied to Latvian individuals and a larger sample to determine whether they have potential clinical relevance and can improve the results.

Mandible condyle development is associated with age. As this maturation takes place around the time an individual reaches legal age, it has the potential to be used as an additional tool for forensic age estimation. Studies on this topic [13,14,15,46] have used CBCT radiographs to evaluate the stage of condylar cortication. In our study, we evaluated condyle cortication on dental panoramic radiographs to determine whether this method can be used to predict chronological age. Logistic regression models showed limited use of condyle cortication stage in this sample. Statistically significant results (*p* < 0.05) were obtained only between condyle maturation stage Types 3 and 1, differences which are already quite obvious radiologically. For now, convincing results for distinguishing between Type 1 and Type 2 and between Type 2 and Type 3 were not observed. The main reason for this outcome could be the limitations in condyle evaluation in dental panoramic radiographs; therefore, this method may only be useful as an additional technique for potential age estimation. Condyles might be evaluated after other age estimation methods have been used. CBCT images offer higher-quality and more accurate views of the condyle, without overlapping structures, compared to dental panoramic radiographs; for this reason, CBCT images are more informative and preferred, as noted in other studies [13,14].

According to the asylum law, border guards can legally request an age estimation procedure if they suspect that an individual has invalid or forged identification papers or is dishonest about their actual age in order to receive various benefits. Frequently, these situations tend to occur around the time that legal age is reached. According to the European Asylum Support Office (EASO)’s practical guide on age assessment [47] and the previously mentioned recommendations by the AGFAD [2], chronological age estimation of a living person includes a dental panoramic radiograph for the assessment of dental maturity, which is usually ordered as part of the legal procedure in relevant situations. During this procedure, it is crucial to apply age estimation methods with higher accuracy and specificity.

The results from studies about age estimation and the I_3m_ index are potentially useful for monitoring migration flow and to identify migrants crossing borders. The flow of migration is worldwide; therefore, it is important to know whether other populations or geographic regions require different cut-off values in order to classify adult vs. non-adults as precisely as possible. The index for each region of the world could be specifically modified and applied in necessary situations.

Although great accuracy was found when obtaining measurements for the I_3m_ index, one of the limitations of this present study is the possibility of measurement errors. Additionally, larger and more equivalent sample sizes would have been preferred. The complex anatomical diversities of the third molars might have influenced the measurements of, for example, the position of the tooth in the longitudinal plane, which made it impossible to perform the index measurements in some radiographs. Another obstacle, which was observed in this study and matched with previous studies on the topic, is third molar agenesis and cases involving extracted third molars [36,48]. As was anticipated, the condyle cortication stage evaluation of dental panoramic radiographs offered varying results, thus showing that further studies need to be conducted on this topic using, preferably, CBCT images. Based on our results regarding the I_3m_ index, future studies need to be conducted to test the potential effectiveness of age estimation by applying the newly proposed cut-off values.

## 5. Conclusions

Within the limits of this study, we can conclude that the I_3m_ index is a reliable age estimation tool, although some modifications could be made to the cut-off value according to gender in Latvian individuals. The condyle cortication stage is a weak tool for chronological age estimation in dental panoramic radiographs. It could be used together with other more reliable age estimation tools.

## Figures and Tables

**Table 1:** Age and gender distribution of the Latvian sample.

Age	Males	Females	Total
14	1	9	10
15	30	40	70
16	18	27	45
17	33	41	74
18	25	80	105
19	36	73	109
20	42	72	114
21	39	87	126
22	18	45	63
Total	242	474	716

**Table 2:** Summary of exclusion criteria.

Exclusion Criteria for I_3m_ Index	Exclusion Criteria for Condyle Cortication
Missing lower-left molar	Low image quality
Low image quality Early crown development stage, roots not present	Images with partially visible condyle area
Lower-left molar with 1 or more than 2 roots	Anatomical structures overlapping
Signs of pathological situations or genetic disorders	Signs of TMJ disorders
Anatomical structures overlapping	Sings or suspicion of genetic disorders

**Table 3:** Binomial logistic regression model fit measures of male, female, and both genders.

Model Type	AUC	AIC	X^2^ (df)	*p*
Male	0.95	87.5	128 (3)	<0.001
Female	0.89	204	114 (3)	<0.001
Both genders	0.92	297	244 (3)	<0.001

**Table 4:** The performance of the logistic regression model as a binary classifier.

Model Type	Accuracy	Specificity	Sensitivity
Male	0.90	0.94	0.82
Female	0.87	0.95	0.55
Both genders	0.89	0.94	0.71

**Table 5:** Logistic regression model coefficients.

Model Type	Predictor	Estimate	Z	*p*	OR	95% CI of OR	Cut-Off Point
Male							
	I_3m_ index	0.11	4.48	<0.001	1.11	1.06–1.17	0.095
	Condyle						
	Type 2–Type 1	−16.6	−0.01	0.992	0	0–inf	
	Type 3–Type 1	−19.7	−0.01	0.990	0	0–inf	
Female							
	I_3m_ index	0.05	4.20	<0.001	1.05	1.02–1.07	0.150
	Condyle						
	Type 2–Type 1	−0.07	−0.06	0.951	0.93	0.08–10.40	
	Type 3–Type 1	−2.61	−2.13	0.033	0.07	0.01–0.81	
Both genders							
	I_3m_ index	0.06	5.90	<0.001	1.06	1.04–1.08	0.095
	Condyle						
	Type 2–Type 1	−1.34	−1.27	0.205	0.25	0.03–2.14	
	Type 3–Type 1	−4.24	−3.88	<0.001	0.01	0.002–0.123	

**Table 6:** Various cut-off values proposed in previously published studies.

Author (year)	Country	Recommended I_3m_ Cut-Off Value Males	Recommended I_3m_ Cut-Off Value Females	Improves
Różyło-Kalinowska et al. (2018) [21]	Poland	0.07	0.07	Specificity for both genders
Dardouri et al. (2016) [45]	Libya	0.09	0.09	Sensitivity for both genders
Balla et al. (2022) [44]	India	0.17	0.17	Sensitivity and accuracy for both genders
Goetten et al. (2022) [23] Sartori et al. (2024) [25]	Northern region of Brazil Southern region of Brazil	0.08 0.06	0.12 0.13	Accuracy for females Accuracy for both genders

## Data Availability

The data presented in this study are available upon request from the corresponding author.

## Abbreviations

The following abbreviations are used in this manuscript:

I_3m_	Third molar maturity index;
CBCT	Cone beam computed tomography;
RSU	Riga Stradins University;
SI	Institute of Stomatology.
